# Nerve ultrasound characterizes AMN polyneuropathy as inhomogeneous and focal hypertrophic

**DOI:** 10.1186/s13023-018-0939-7

**Published:** 2018-11-03

**Authors:** Tim W. Rattay, Jennifer Just, Benjamin Röben, Holger Hengel, Rebecca Schüle, Matthis Synofzik, Anne S. Söhn, Natalie Winter, Nele Dammeier, Ludger Schöls, Alexander Grimm

**Affiliations:** 1 Center for Neurology, and Hertie-Institute for Clinical Brain Research, University of Tübingen, Hoppe-Seyler-Straße 3, 72076 Tübingen, Germany; 2 German Center of Neurodegenerative Diseases (DZNE), Tübingen, Germany; 3 Institute of Medical Genetics and Applied Genomics, Tübingen University Hospital, Tübingen, Germany

**Keywords:** Adrenoleukodystrophy, Adrenomyeloneuropathy, X-ALD, Nerve conduction study, High resolution nerve ultrasound, Very long chain fatty acids, Ultrasound pattern sum score, Peripheral neuropathy

## Abstract

**Objective:**

High-resolution nerve ultrasound (HRUS) is a painless tool to quickly evaluate peripheral nerve morphology in vivo. This study set out to characterize peripheral nerve involvement in X-linked adrenomyeloneuropathy (AMN) by HRUS.

**Methods:**

Thirteen adults with genetically proven AMN were examined using the Ultrasound pattern sum score (UPSS) to evaluate morphological abnormalities of peripheral nerves, vagal nerves, as well as cervical nerve roots. Ultrasound results were correlated with clinical findings and nerve conduction studies.

**Results:**

UPSS was increased in six out of 13 patients. Nerve enlargement was mostly inhomogeneous and regional. The median, ulnar, and vagal nerves presented with more prominent alterations than nerves of the lower limbs. The proximal-to-distal ratio was significantly enlarged for the median nerve. HRUS findings matched nerve conduction studies, but identified one patient with enlarged nerves and yet normal conduction velocities. Sonographic findings did not correlate with disease duration or disease severity as assessed by the spastic paraplegia rating scale.

**Conclusion:**

HRUS reveals significant multifocal regional nerve swellings with reduced echo intensity as the morphological equivalent of electrophysiological peripheral nerve affection in AMN patients. Ultrasound and NCS characteristics in AMN seem to differ from other demyelinating neuropathies like CIDP or CMT1a.

**Trial registration:**

German clinical-trial-register (DRKS) (DRKS-ID 00005253) Registered 15 October 2013.

**Electronic supplementary material:**

The online version of this article (10.1186/s13023-018-0939-7) contains supplementary material, which is available to authorized users.

## Background

High-resolution ultrasound (HRUS) is an inexpensive, quick, and comfortable tool to screen for nerve cross-sectional area (CSA) changes. These changes range from focal to multifocal, or homogenous enlargements [1] even without electrophysiological changes. Despite obvious advantages of HRUS to identify and evaluate structural peripheral nerve alterations, no data for X-linked adrenoleukodystrophy (X-ALD) exists. X-ALD is the most common peroxisomal disorder worldwide [2]. The disease is caused by impaired peroxisomal beta-oxidation due to mutations in the *ABCD1* gene [3] on the X-chromosome leading to an accumulation of very-long chain fatty acids (VLCFA) in plasma as well as tissues including white matter of the brain, spinal cord, and adrenal cortex. Patients with adult onset usually develop the adrenomyeloneuropathy (AMN) phenotype characterized by progressive spastic paraparesis with bladder disturbance, sensory ataxia with impaired vibration sense, pain in the legs and in male patients, adrenal failure. Nerve conduction studies (NCS) mostly reveal multifocal demyelination [4] with lower extremities most frequently and most severely affected [5]. Central findings [6] include prolonged central somatosensory conduction and prolonged central motor conduction.

## Methods

### Design, setting and participants

Between 04/2016 and 01/2018 this observational cross-sectional study recruited a consecutive series of 14 genetically proven AMN patients from our leukodystrophy clinic. Patient #14 was excluded prior to data analysis due to a confounding additional diagnosis of neurofibromatosis type 1 [7]. Clinical characteristics and genetic results of all patients are presented in Additional file 1: Table S1. For the control group 13 healthy age-, sex-, and BMI-matched controls were recruited from medical staff and further individuals without signs of neuromuscular disorders. The study was registered with the German clinical-trial-register (DRKS-ID 00005253) and approved by the local ethic committee (Tübingen 702/2015BO2). Written informed consent was obtained from all participants.

### High-resolution nerve ultrasound

B-mode ultrasound studies were performed by well-experienced sonographers (AG, TWR, ND and NW, > 3 years of experience and > 1000 examinations) blinded to the clinical examination with a high-resolution probe (9–16 MHz broad band linear probes, TE7, Mindray company, Darmstadt) of easily accessible peripheral nerves [median nerve (MN), ulnar nerve (UN), radial nerve (RN), tibial nerve (TN), fibular nerve (FN), sural nerve (SN)], vagal nerve (VN), and the C5 and C6 nerve roots of the brachial plexus. Nerve CSA was determined at predefined landmarks [8, 9] and then scored according to the Ultrasound pattern sum score (UPSS). The homogeneity score (HS) [10] was evaluated as described before for hereditary neuropathies. Additionally the classification described by Padua et al. for distinct nerve aspects including the echointensity of the nerves as either hypoechoic enlarged (Class 1), hyperechoic enlarged (Class 2), or not enlarged (Class 3) was evaluated [11]. Echointensity was quantified semiquantitatively: if nerve overall aspect was comparable to vessel lumen it was rated hypoechoic (Class 1) and if it was similar to lymph node aspects it was rated hyperechoic (Class 2). To check for proximal-to distal predominance of nerve pathology a ratio (CSA nerve proximal/CSA nerve distal) in median, ulnar and tibial nerves was calculated and then compared between patients with and without neuropathy.

### Clinical assessment / electrophysiology

Standard assessment included nerve conduction studies (NCS) of the UN (motor and sensory), the TN (motor), and the SN (sensory). Nerve conduction studies were recorded on the same body side on which ultrasound examinations were performed using standard conditions described in [12]. A standardized clinical examination including the Spastic Paraplegia Rating Scale (SPRS) [13] and/or the Scale for the Assessment and Rating of Ataxia (SARA) [14] was performed by a movement disorder specialist. In addition, sensory- and/or motor evoked potentials were recorded in a subset of patients.

### Statistical analysis

For statistical analysis, IBM SPSS Statistics, version 24 (Chicago, IL) was used. Group differences of biographic and clinical data were assessed when normally distributed by t-test with Bonferroni correction and if not normally distributed by Mann-Whitney-test with Bonferroni correction. The corresponding statistical test is specified in the results section. Regression analysis was performed to evaluate correlations between ultrasound and nerve conduction measures. Receiver operating characteristics (ROC) curve analysis identified boundary values to differentiate demyelinating changes from normal findings. In all tests a *p*-value < 0.05 (two-sided testing) was considered to be statistically significant.

## Results

On examination, clinical signs of potential peripheral neuropathy were common. 92% of patients had afferent ataxia, 85% showed distally pronounced weakness of the lower limbs, and 62% had malleolar pallhypaesthesia. Neither nerve conduction studies (NCS) nor ultrasound findings correlated with clinical signs of neuropathy, with disease duration, or disease severity as assessed by the SPRS [13]. Detailed clinical as well as genetic data of all patients is presented in Additional file 1: Table S1.

**Fig. 1:**
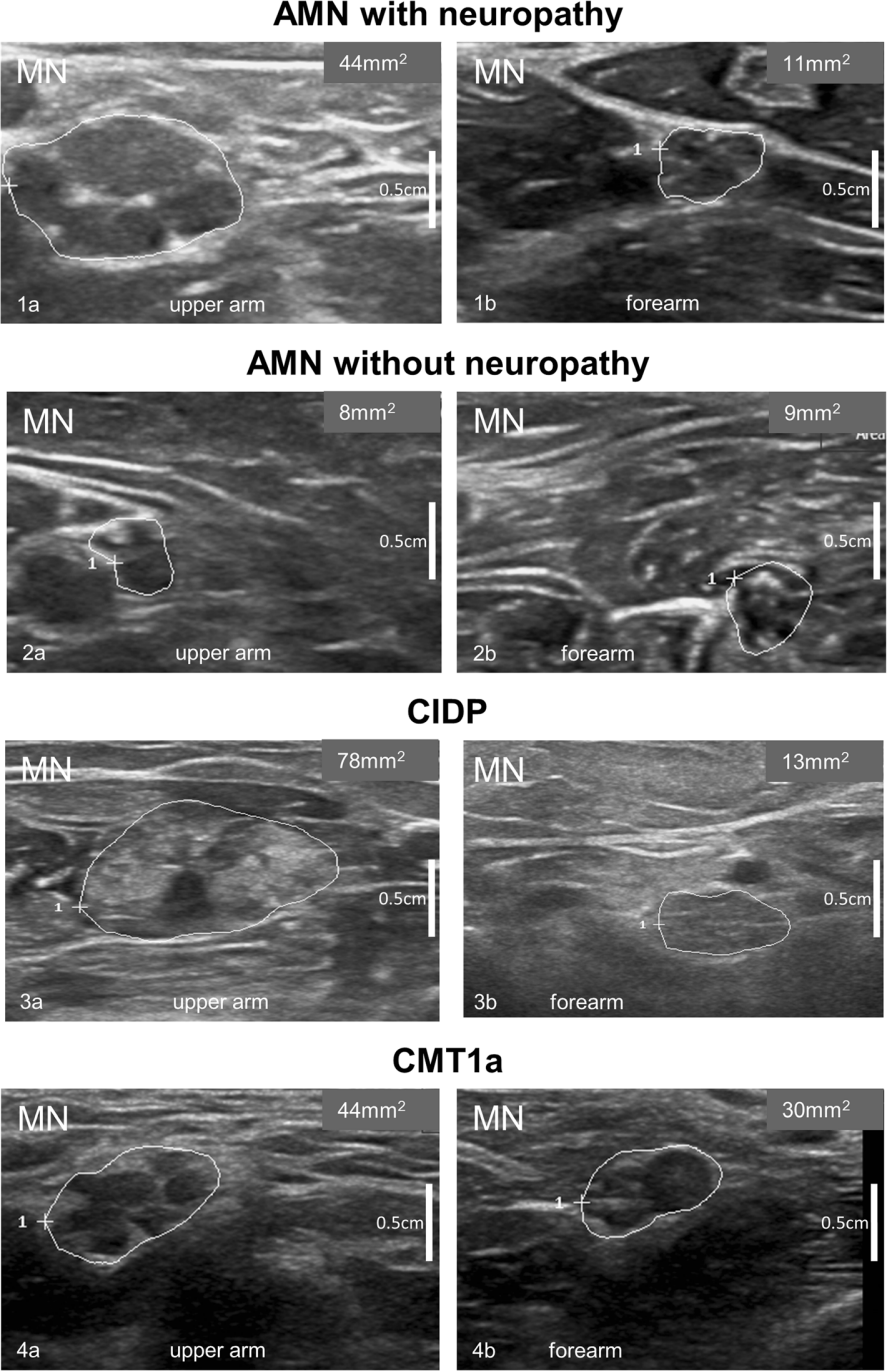
Representative ultrasound images of the median nerve (MN) in different diseases / disease states are shown per row. The left column (pictures labeled with an (**a**)) depicts cross sections of the upper arm (UA) and in the right column (**b**) cross sections of the forearm (FA). All ultrasound cross sections were recorded according to the UPSS protocol as previously described [8, 9]. All ultrasound pictures are presented at the same resolution and are therefore comparable in size (scale bar indicates 0.5 cm). In the first row (Ia&b) the inhomogenously enlarged median nerve of a patient with AMN and demyelinating polyneuropathy is seen (Class 1 according to [11]), with a cross-sectional area (CSA) of 44 mm^2^ in the UA (Ia) and 11mm^2^ in the FA – (Ib). Images of an AMN patient without neuropathy (Class 3, pictures IIa&b) are presented in the second row. The CSA of the MN was 8mm^2^ in the UA (IIa) and 9mm^2^ in the FA (IIb). CSA values of AMN patients without electrophysiologically proven peripheral neuropathy (PNP) correspond to normal values of healthy controls as published before [8]. For comparison purposes, we added representative pictures of a chronic inflammatory demyelinating polyneuropathy (CIDP) patient (Class 2) in the thirds row (IIIa & b) and of a Charcot-Marie-Tooth type 1a (CMT1a) patient (IVa & b) in the fourth row. The CIDP patient shows an inhomogeneously enlarged median nerve (78mm^2^ in the UA (IIIa) and 13mm^2^ in the FA (IIIb)) which is hyperechoic due to more perifascicular tissue. Nerve segments of the median nerve of a CMT1a patient (IVa & b) are in contrast homogeneously enlarged with 44mm^2^ in the UA (IVa) and 30mm^2^ in the FA (IVb) without significant changes in echointensity. The CSA in CMT1a equals the 3-4fold of known normal values in healthy adults

**Fig. 2:**
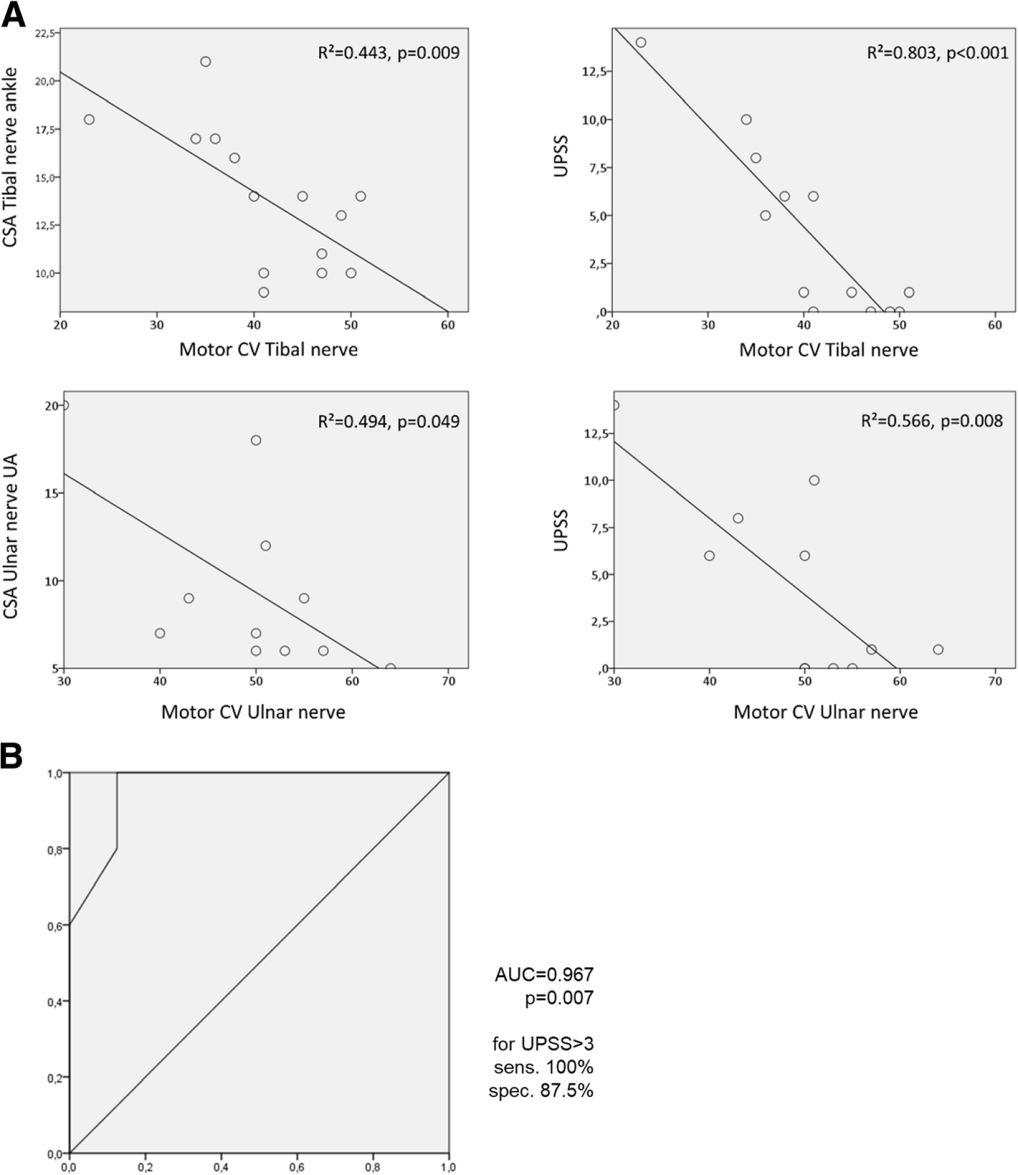
(**a**) Scatter plot showing an inverse correlation of motor conduction velocity (CV) of tibial and ulnar nerves with the cross-sectional area (CSA) of the corresponding nerves. In addition, CVs correlate inversely with the ultrasound pattern sum score (UPSS): the higher the UPSS (indicating overall nerve enlargement), the lower the CV. (**b**) Receiver Operating Characteristic (ROC) curve analysis for the ultrasound pattern sum score (UPSS) (summarizing all enlarged nerve segments) to differentiate AMN with and without polyneuropathy: A score > 3 points is highly sensitive and specific for AMN with demyelinating neuropathy

Nerve ultrasound found CSA of peripheral nerves to be enlarged in 6/13 patients. Nerve enlargement was mostly inhomogeneous or regional (Fig. 1 (Ia&b) and Table 1). CSA enlargement was most prominent in the proximal upper extremities (median and ulnar nerve) and the vagal nerve. It was also found in the C5 root, the sural nerve, and the distal segment of the tibial nerve (Table 1). The proximal to distal ratio showed significant differences for the median nerve (t-test with Bonferroni *p* = 0.021 with larger ratios in AMN with neuropathy (median ratio 2.0, range 1.5–3.6) compared to patients without neuropathy (median ratio 1.3, range 0.9–1.5) and controls (median ratio 1.3, range 1.0–1.7)), with no significant findings in the ulnar and tibial nerve. NCS revealed significantly reduced motor nerve conduction velocity (CV) in 5/13 patients with AMN ranging from 23 to 42 m/s in the UN and from 20 to 36 m/s in the TN while in eight AMN patients NCS were unremarkable (Additional file 1: Table S1). Interestingly, nerve ultrasound findings were abnormal in all AMN patients with electrophysiological indicators of demyelinating peripheral neuropathy. Vice versa, ultrasound measurements were normal (Fig. 1 – pictures IIa&b) in all but one AMN patient (#3) without neuropathy in NCS; in this patient (#3) CSA enlargement was restricted to the lower limbs (Additional file 1: Table S1). CSA showed close inverse correlation with motor nerve CV of the tibial and ulnar nerve in all patients (Fig. 2a). CSA did not correlate significantly with sensory nerve CVs of the ulnar (*p* = 0.142) and sural nerve (*p* = 0.173). The ultrasound pattern sum score (UPSS) as overall nerve enlargement score, its subscores and the HS revealed significant differences between AMN patients with and without neuropathy.

**Table 1:** Ultrasound findings in patients with AMN

	n (# of subjects)	Median nerve	Ulnar nerve	Radial nerve	Fibular nerve	Tibial nerve	Sural nerve	Vagal	C5 / C6	UPSS	UPSA	UPSB	UPSC	HS
AMN with demyelinating neuropathy	6	14*/14*/9/15*	12/10*/8*	3	11/2	28/17*	4*	4*	4.9*/2.7	8***	5**	2***	1**	2**
AMN without neuropathy	7	10/10/8/10	7/7/6	2	8/2	21/12	2	2	3.4/2.4	0.5	0.5	0	0	0
Control	13	9/10/7/10	7/9/6.5	2	8/2	24.5/10	2	2	3.7/2.4	0	0	0	0	0

*AMN* = Adrenomyeloneuropathy, *C5* / *C6* = cervical root 5 and 6; *CSA* = cross sectional area, *PNP* = peripheral neuropathy; *HS* = homogeneity score

*UPSS* = ultrasound pattern sum score; *UPSA* = part A of the UPSS, *UPSB* = part B of the UPSS, *UPSC* = part C of the UPSS

*: *p* < 0.05, **: *p* < 0.01, ***:*p* < 0.001 when compared to the healthy controls and AMN without neuropathy

Median values of cross-sectional area measurements are provided for different sections of peripheral nerves (from proximal to distal in mm^2^) and for cervical nerve roots (diameter in mm). Significant enlarged nerves are marked in bold

By ROC-curve analysis a cut-off value > 3 in the UPSS was evaluated to have the best sensitivity and specificity to differentiate between AMN patients with and without electrophysiological evidence of polyneuropathy (Fig. 2b).

The HS showed significantly larger results in AMN with neuropathy compared to those without (*p* = 0.004) and the healthy controls (*p* = 0.002) tested by t-test with Bonferroni correction. Although median values of CSA and UPSS were slightly higher in males than in females, there was no significant gender difference. Similarly, no effect of age, height, or weight on ultrasound data was detected (all parameters tested with Mann-Whitney-test). All patients with AMN and nerve enlargement showed reduced echointensity of the fascicles corresponding to Class 1 pattern described by Padua and colleagues [11] (Fig. 1, Ia and b). All but one patient with AMN and no electrophysiological neuropathy revealed Class 3 pattern.

## Discussion

In our AMN cohort significant electrophysiological neuropathy was found in about 50% of cases equally affecting males and females. This is less than the clinical findings suggested but central affection can lead to an overestimate of peripheral nerve involvement. If present, AMN-associated neuropathy shows demyelinating characteristics in NCS as previously described [4]. HRUS revealed AMN neuropathy being accompanied by regional and predominantly proximal hypertrophy, particularly of the median nerve and radices. We propose that increased nerve CSA represents the primary morphological equivalent of AMN neuropathy. When comparing HRUS findings in AMN to other demyelinating polyneuropathies like Charcot-Marie-Tooth type 1a (CMT1a) or chronic inflammatory demyelinating polyneuropathy (CIDP), striking differences stand out (see Fig. 1): CMT1a typically leads to homogeneous nerve enlargement in HRUS, homogeneous decreased CV in motor and sensory NCS, and onion-bulb formations in histopathology caused by chronic de- and re-myelination. Both HRUS scores, UPSS and the homogeneity score (HS) are obviously higher in CMT1a (as described in [10]) than in AMN. In CMT1a the echointensity is reduced with swollen fascicles to the best of our knowledge and thus would be classified as Class 1 according to Padua et al.; however, we must admit, that this classification has never been used for hereditary neuropathies so far. In CIDP, HRUS depicts rather inhomogeneous nerve enlargement with predominance in roots and nerve sections at the upper arm. In contrast to AMN (Padua Class 1 pattern according to [11]), increased nerve echo as a potential ultrasound sign of intraneural fibrosis might be a typical finding in many CIDP patients, particularly in those with long disease duration [11, 15]. Härtig and colleagues correlated histology of those patients with increased echointensity (Padua Class 2 pattern) and demonstrated more axonal damage in those nerve biopsies. NCS typically finds regionally restricted slowing of CV with prolonged F-wave latency, temporal dispersion or conduction blocks in CIDP. Histopathology reveals onion-bulb formations [2] in combination with inflammation, edema, fibrosis and axonal damage [16].

Taken together, differences can be summarized as following (compare Fig. 1):

**CMT1a**: homogeneous nerve enlargement and homogeneous CV slowing.

**CIDP**: inhomogeneous, regionally restricted nerve enlargement with often increased echo intensity and inhomogeneous CV slowing.

**AMN**: inhomogeneous, regionally restricted nerve enlargement with decreased echo intensity and homogeneous CV slowing.

Due to the limited number of nerve biopsies and the restriction to the distal sural nerve of patients with AMN it is difficult to define the histopathological basis of HRUS abnormalities in AMN. In contrast, skin biopsy data showing lack of small nerve fibers in patients with AMN is available [17]. The predominant involvement of proximal nerve segments of the upper extremities (particularly the median nerve) is noteworthy, as similar findings have been described for acquired inflammatory neuropathies, i.e. CIDP [18] or MMN [19] and even inherited neuropathies, i.e. Friedreich’s ataxia [20] or familial amyloidosis [21]. The pathophysiological background of this finding has not been clarified until now, but proximal nerve segments might be more vulnerable than distal ones. Thickening of nerves might be a sign of concomitant inflammation – as in CIDP – which could even pinpoint to therapeutic steps, however this needs to be clarified by additional investigations, i.e. CSF analysis, MRI with gadolinium, biopsies or post mortem tissue. Another explanation could be a type of hypertrophic remodeling, which has already been discussed before for inflammatory and inherited neuropathies [22].

Predisposing factors of neuropathy in AMN are not known [2] and the evolution of AMN neuropathy is still enigmatic. There were no correlations with gender, age, height, weight, or disease severity. It is unclear to which extent the demyelinating process found in our case series results in axonal damage during further disease progression. This is well established for other hereditary demyelinating neuropathies like CMT1a. Furthermore, it is unclear whether structural nerve changes as recognized in HRUS precede electrophysiological changes or if they occur later (as it is potentially the case in patient #3). Longitudinal follow-up with repeated ultrasound examination can help to answer these questions. Interestingly, nerve swellings are more prominent in the upper extremities. As ultrasound changes do not correlate with disease duration, age, or disease severity there is no hint that this reflects secondary atrophy of distal parts of the nerves after initial swelling. Better understanding of the development of AMN neuropathy is needed and HRUS may aid or guide histopathological understanding to further advances.

## Conclusion

Nerve ultrasound is a reliable, painless, and easily accessible tool to characterize peripheral nerve involvement in AMN patients. Our findings have profound practical implications because neuropathy in AMN patients is characterized by multifocal proximal nerve enlargements with reduced echo intensity in high resolution ultrasound and homogeneous reduction of conduction velocity in nerve conduction studies. Ultrasound and NCS characteristics in AMN seem to differ from other demyelinating neuropathies. As ultrasound is a fast examination technique that does not cause discomfort to the patients it has compliance advantages compared to electrophysiological examinations. Long-term follow-up with HRUS will help to disclose the evolution of peripheral neuropathy in patients with AMN.

## Additional file

Additional file 1: **Table S1.** Detailed clinical data of all AMN cases. (DOCX 24 kb)

## Abbreviations

AMN: Adrenomyeloneuropathy
CIDP: Chronic inflammatory demyelinating polyneuropathy
CMT1a: Charcot-Marie-Tooth type 1a
CSA: Cross-sectional area
CV: Conduction velocity
FA: Forearm
FN: Fibular nerve
HRUS: High resolution ultrasound
HS: Homogeneity score
MN: Median nerve
NCS: Nerve conduction study
PNP: Peripheral neuropathy
RN: Radial nerve
ROC: Receiver Operating Characteristic
SARA: Scale for the rating of ataxia
SN: Sural nerve
SPRS: Spastic paraplegia rating scale
TN: Tibial nerve
UA: Upper arm
UN: Ulnar nerve
UPSS: Ultrasound pattern sum score
VLCFA: Very-long chain fatty acids
VN: Vagal nerve
X-ALD: X-linked adrenoleukodystrophy
