# Supplementary material for: Nerve ultrasound characterizes AMN polyneuropathy as inhomogeneous and focal hypertrophic
Source: Orphanet J Rare Dis. 2018 Nov 3;13:194. doi: 10.1186/s13023-018-0939-7 (PMC6215661; doi:10.1186/s13023-018-0939-7)
Supplement: Supplementary file 1 — Table S1. Detailed clinical data of all AMN cases. (DOCX 24 kb) [file 13023_2018_939_MOESM1_ESM.docx]

**Additional file 1: Table S1:**

**Detailed clinical data of all AMN cases**

| **Patient #** | **ABCD1 mutation** | **Ethnicity** | **Sex** | **Age Of Onset [years]** | **Age at examination [years]** | **Bladder dysfunction** | **Adrenal insufficiency** | **Spasticity** | **Gait ataxia** | **Ddistally pronouced weakness / paresis** | **Hypesthesia / paraesthesia** | **Malleolar pallhypesthesia** | **Impaired  proprioception** | **SARA score** | **SPRS score** | **High resolution ultrasound**  [Median nerve CSA (mm^2^) without wrist measurement] |  | **Nerve conduction studies** | **Very long chain fatty acids** | **Prolonged CMCT  (UE/LE)** | **Abnormal SEP  (UE/LE)** |
| --- | --- | --- | --- | --- | --- | --- | --- | --- | --- | --- | --- | --- | --- | --- | --- | --- | --- | --- | --- | --- | --- |
| 1 | c.623_624del, p.V208Gfs*92 | Russian | m | 30 | 51 | Urge | yes | tetra- | yes | LE | no | no | no | - | 27 | CSA ↑  [41/30/13] |  | s+m NCV↓ | ↑ | UE/LE | UE/LE |
| 2 | c.1816T>C, p.S606P | German | f | 12 | 54 | Urge | no | para- | no | LE | no | no | no | - | - | Normal |  | Normal | ↑ | normal | normal |
| 3 | c.2030C>A, p.G677D | German | f | 53 | 64 | Urge | no | para- | yes | LE | LE | yes | no | - | 16 | CSA LE ↑  [20/13/10] |  | Normal | - | LE | - |
| 4 | c.887A>G, p.Y296C | German | m | 41 | 64 | Urge | no | tetra- | yes | LE | m | yes | LE | - | 23 | CSA ↑  [14/14/8] |  | s+m NCV↓ | ↑ | - | - |
| 5 | c1978C>T, p.R660W | German | m | 45 | 55 | voiding | yes | tetra- | yes | LE | no | yes | no | - | 14 | Normal |  | Normal | ↑ | - | LE |
| 6 | c.1661G>A, p.R554H | German | m | 35 | 43 | - | yes | para- | yes | no | no | no | m | 10 | - | Normal |  | Normal | - | - | - |
| 7 | c.411G>T, p.W137C | German | m | 42 | 58 | Urge | no | para- | yes | LE | LE | yes | UE/LE | - | 27 | CSA ↑  [14/16/9] |  | s+m NCV↓ | ↑ | LE | UE/ n.p. |
| 8 | c.1252C>T, p.R418W | German | f | 50 | 60 | Urge | - | para- | yes | LE | no | yes | no | - | 14 | CSA ↑  [13/14/9] |  | s+m NCV↓ | ↑ | UE/LE | - |
| 9 | c.1252C>T, p.R418W | German | m | 32 | 37 | No | - | para- | yes | LE | no | no | no | - | 11 | CSA ↑  [12/12/7] |  | s+m NCV↓ | ↑ | - | - |
| 10 | c.1992G>A, p.W664* | German | m | 50 | 58 | voiding | yes | tetra- | yes | LE | no | no | no | 8 | - | normal |  | s NCV↓ | ↑ | - | UE/LE |
| 11 | c.1992G>A, p.W664* | German | f | 37 | 62 | voiding | no | para- | yes | LE | no | yes | no | - | 22 | normal |  | normal | ↑ | LE | LE |
| 12 | c.1534G>A p.G512S | Italian | m | 6 | 28 | No | yes | para- | yes | no | no | yes | UE/LE | 2 | 3 | normal |  | normal | ↑ | normal | - |
| 13 | c.119_147del, p.L40RfsX145 | Kazakh | f | 40 | 61 | Urge | no | tetra- | yes | LE | UE/LE | yes | no | 6 | 21 | normal |  | normal | ↑* | UE/LE | UE+LE n.p. |
|  |  |  |  |  |  |  |  |  |  |  |  |  |  |  |  |  |  |  |  |  |  |
| 14 | c.1816T>C, p.S606P  **NF1:** c.1186-1G>C (IVS8), p.? | Thai | f | 25 | 42 | Urge | no | tetra- | yes | both | yes | yes | no | - | 19 | typical NF1 findings |  | CB fibular nerve | ↑ | UE/LE | UE normal /LE |

**Abbreviations:** - = missing data, CB=conduction block, CMCT=corticomuscular conduction time, CSA=cross sectional area, dem.=demyelinating, f=female, incr.=increased, LE=lower extremity, m=male, NCV: Nerve conduction velocity; n.p.=not possible, para-=paraspasticity, s.=sensory, SARA=scale for the assessment and rating of ataxia, SEP=sensory evoked potential, s-m.=sensory-motor, SPRS=spastic paraplegia rating scale, tetra-=tetraspasticity, UE=upper extremity, UMN=upper motor neuron; ↓: reduced. ↑: increased. **↑*** = only C24 very long chain fatty acids elevated, C26 normal.
